# Resistance to the larvicide temephos and altered egg and larval surfaces characterize salinity-tolerant *Aedes aegypti*

**DOI:** 10.1038/s41598-023-35128-1

**Published:** 2023-05-19

**Authors:** Kokila Sivabalakrishnan, Murugathas Thanihaichelvan, Annathurai Tharsan, Thamboe Eswaramohan, Punniamoorthy Ravirajan, Andrew Hemphill, Ranjan Ramasamy, Sinnathamby N. Surendran

**Affiliations:** 1 Department of Zoology, Faculty of Science, University of Jaffna, Jaffna, 40000 Sri Lanka; 2 Department of Physics, Faculty of Science, University of Jaffna, Jaffna, 40000 Sri Lanka; 3 Department of Infectious Diseases and Pathobiology, Vetsuisse Faculty, Institute of Parasitology, University of Bern, Länggass-Strasse 122, 3012 Bern, Switzerland

**Keywords:** Ecology, Zoology

## Abstract

*Aedes aegypti*, the principal global vector of arboviral diseases and previously considered to oviposit and undergo preimaginal development only in fresh water, has recently been shown to be capable of developing in coastal brackish water containing up to 15 g/L salt. We investigated surface changes in eggs and larval cuticles by atomic force and scanning electron microscopy, and larval susceptibility to two widely-used larvicides, temephos and *Bacillus thuringiensis,* in brackish water-adapted *Ae. aegypti*. Compared to freshwater forms, salinity-tolerant *Ae. aegypti* had rougher and less elastic egg surfaces, eggs that hatched better in brackish water, rougher larval cuticle surfaces, and larvae more resistant to the organophosphate insecticide temephos*.* Larval cuticle and egg surface changes in salinity-tolerant *Ae. aegypti* are proposed to respectively contribute to the increased temephos resistance and egg hatchability in brackish water. The findings highlight the importance of extending *Aedes* vector larval source reduction efforts to brackish water habitats and monitoring the efficacy of larvicides in coastal areas worldwide.

## Introduction

*Aedes aegypti,* the principal global vector of arboviral diseases including dengue, chikungunya, yellow fever and Zika, has been widely regarded to lay eggs and undergo preimaginal development only in fresh water (FW)^1–3^. Larval source reduction measures that involve applying larvicides and eliminating or reducing its preimaginal FW habitats are globally important for controlling arboviral diseases^1–4^. The organophosphate temephos is the most commonly used larvicide for this purpose, while *Bacillus thuringiensis* is also utilized as a biological larvicide^1–4^. Dengue is the most prevalent arboviral disease in the world with > 100 million annual cases^1–3^. Dengue-endemic Sri Lanka and its northern peninsular Jaffna district (Supplementary Fig. 1), recorded 105,049 and 8261 dengue cases respectively in 2019^5–7^, but the numbers decreased sharply and temporarily in 2020 and 2021 during COVID-19-related movement restrictions^7,8^. *Aedes aegypti* and the secondary arboviral vector *Aedes albopictus* were recently shown to lay eggs and undergo preimaginal development in brackish water (BW) habitats of up to 15 g/L salt in the 1100 km^2^ Jaffna peninsula^6,9–12^, with FW, BW and saline water respectively defined as containing < 0.5, 0.5–30 and > 30 g/L salt^9^. The preimaginal development of the two *Aedes* vectors in BW has since been documented in other countries^13–18^. BW habitats of *Ae. aegypti* in the Jaffna peninsula include water collections in beach debris and fishing boats^9^, coastal domestic wells^6,10,11^, discarded containers in mangrove swamps^11^, stagnant drains^12^ and household containers in coastal areas ^6,11,12^. BW-derived *Ae. aegypti* and *Ae. albopictus* larvae in the Jaffna peninsula are more salinity-tolerant, possessing higher LC_50_ for salt than FW-derived larvae from the Jaffna peninsula and the Sri Lankan mainland^9,19^. Adaptation to BW in *Ae. aegypti* larvae is characterized by inheritable changes^19^, alterations in the osmoregulatory anal papillae of larvae^20^, changes in gene expression and cuticle protein composition in fourth instar (L4) larvae^21^, and ultrastructural changes within L4 and adult cuticles observed by transmission electron microscopy^21^. We proposed that the changes observed in larval cuticles may make salinity-tolerant *Ae. aegypti* larvae less susceptible to important larvicides^21^.

Adaptation of *Ae. aegypti* and *Ae. albopictus* and other typically FW mosquito vectors, such as the malaria vectors *Anopheles culicifacies*^22^ and *Anopheles stephensi*^23^, to develop in BW can increase the transmission of vector-borne diseases in coastal areas because BW habitats are globally neglected in larval source reduction strategies^24–29^. Increasing salinization of ground water in coastal areas due to rising sea levels and unsustainable extraction of ground water, which is evident in the Jaffna peninsula^28^, contributes to an expansion of BW habitats for mosquito vectors^24–29^. Approximately 10% of the global population live in coastal areas making this is a worldwide problem that is more severe in locations with a high ratio of coastline to land area^24–26^.

Scanning electron microcopy (SEM) has been used to study the surface topography of eggs in FW *Ae. aegypti* and *Ae. albopictus*^30^, and other mosquito species^31–35^. Atomic force microscopy (AFM) has been utilized to characterize surface hardness (or elasticity) and surface roughness (or topography) of materials in physical sciences but has only rarely been applied to biological specimens^36^. We show that salinity-tolerant *Ae. aegypti* are characterized by smaller eggs, surface changes in larval cuticles and eggs detected by AFM and SEM, as well as significantly reduced larval susceptibility to temephos, but not *Bacillus thuringiensis,* compared to FW *Ae. aegypti*. The findings have important implications for the control of mosquito vector-borne diseases.

## Results

### Salinity tolerance and reproductive compatibility of BW and FW *Aedes aegypti* laboratory colonies

We previously showed that the LC_50_ for salt in the L1 to adult transformation was significantly higher for BW (15.6 g/L) than FW (12.2 g/L) in the 2nd generation (G2) of colonies established with *Ae. aegypti* collected from BW and FW field habitats in the Jaffna peninsula and then maintained respectively in 10 g/L salt BW and 0 g/L salt FW in the laboratory^19^. Because later generations of the Jaffna BW (designated JBW) and FW (designated JFW) laboratory colonies were used in the present work, we verified that the G66 JBW laboratory colony retained significantly (*p* < 0.05) higher LC_50_ (16.0 g/L) than the G66 JFW laboratory colony (LC_50_ of 11.6 g/L) for the L1 to adult transition (details in Supplementary Table 1). A G17 FW *Ae. aegypti* colony derived from Nawalapitiya in the central highlands of mainland Sri Lanka where BW pre-imaginal habitats are completely absent (Supplementary Fig. 1), designated NFW, had an LC_50_ (11.2 g/L) that was not significantly different from the JFW colony but significantly lower than the JBW colony for the L1 to adult transition (Supplementary Table 1).

We previously reported that the G8 JBW and JFW laboratory colonies were reproductively compatible^19^. We verified that G74 and G75 JBW and JFW colonies retained reproductive compatibility in reciprocal crosses (Supplementary Table 2). However, eggs laid by JFW females in the crosses showed reduced hatchability and fewer adults developing from eggs in 10 g/L salt BW than FW (Supplementary Table 2), suggesting that these properties were maternally inherited.

### Morphology of eggs and L4 of JBW and JFW *Aedes aegypti* laboratory colonies characterized by light microscopy

JBW eggs were smaller than JFW eggs when examined by light microscopy at 100 × magnification. The dimensions of one hundred eggs from ten iso-females (comprising ten eggs per iso-female) were measured from the G62, G66 and G70 generations of JBW and JFW colonies. The maximum lengths and widths of JBW eggs were significantly smaller than JFW eggs at *p* < 0.05 (Supplementary Fig. 2). Light microscopic examination at 10 × magnification of mid-L4 bodies of G73 JFW and G73 JBW colonies showed no detectable differences (Supplementary Fig. 2).

### Egg surface changes characterize salinity tolerance in *Aedes aegypti* laboratory colonies

**Figure 1:**
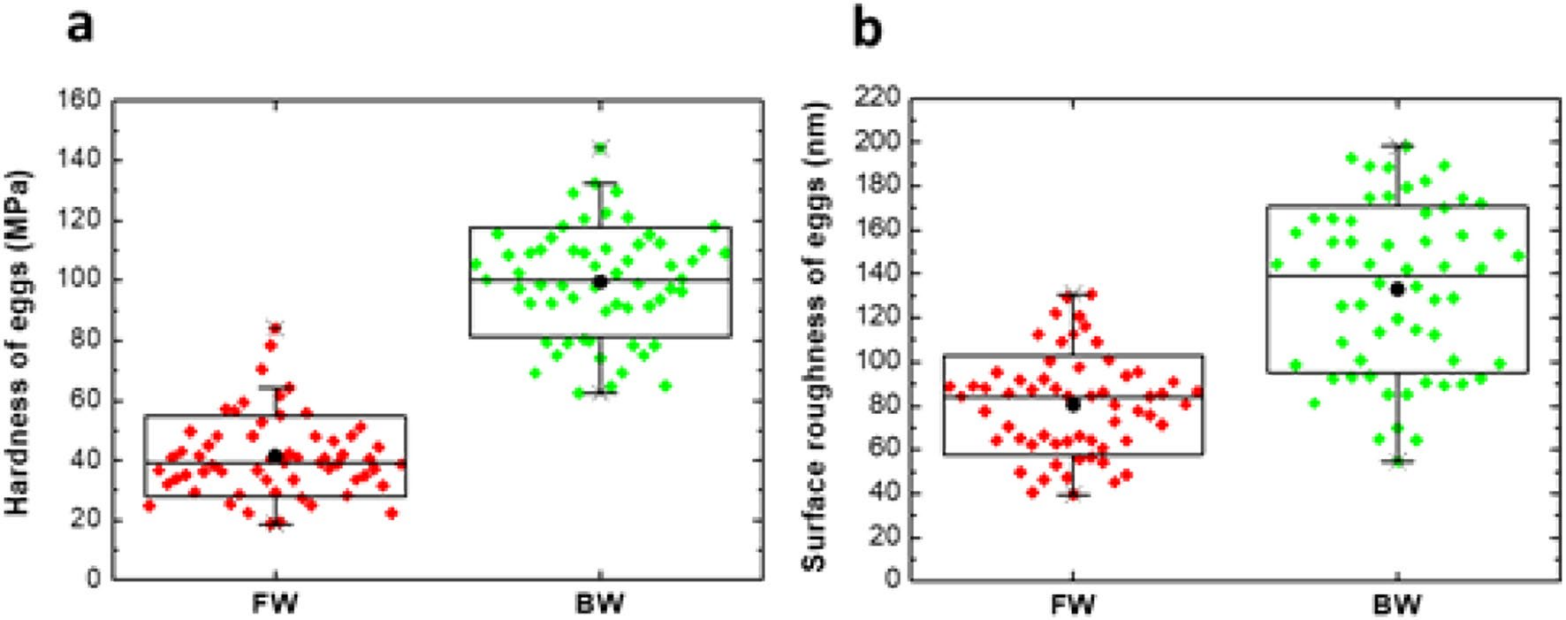
Surface hardness in MPa (**a**) and roughness in R_RMS_ (**b**) of G70 JFW (labelled as FW in the figure) and JBW (labelled as BW in the figure) eggs from laboratory colonies. MPa-modulus of elasticity in mega Pascals. R_RMS_-root mean square average height deviations in nm. The box-and-whiskers plots show the lower and upper quartiles and the median. Black solid circles show the mean.

The surface hardness and roughness determined by AFM in five eggs laid by each of six randomly selected females from the G70 JBW and JFW colonies are shown in Fig. 1.

The results demonstrated that JBW colony eggs had significantly greater (*p* < 0.0001) surface hardness (modulus of elasticity in MPa, mean ± standard deviation of JFW = 41 ± 13 and JBW = 100 ± 24;) and surface roughness (R_RMS_ in nm, mean ± standard deviation of JFW = 80 ± 8 and JBW = 133 ± 14;) than JFW colony eggs.

**Figure 2:**
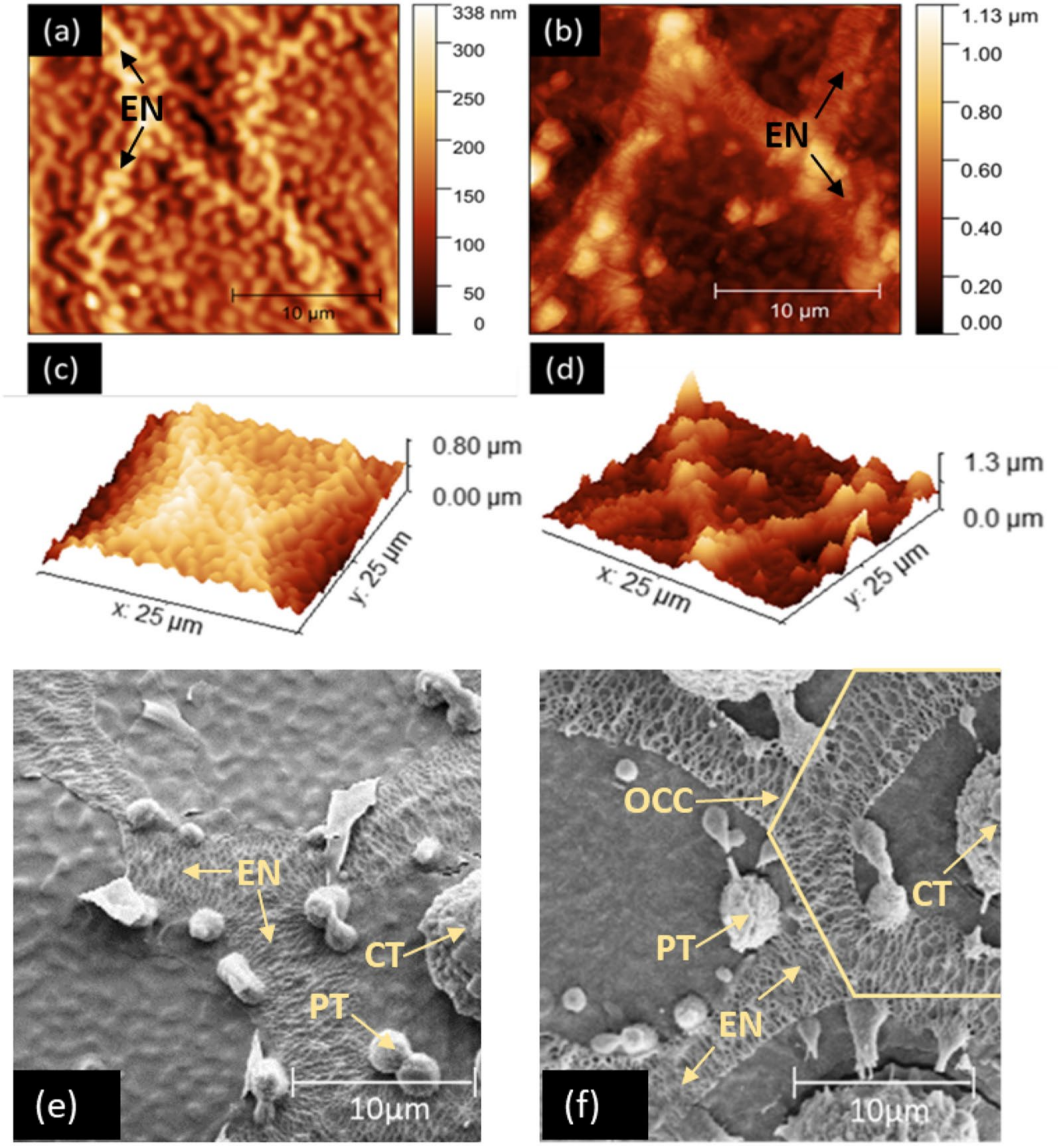
Surface topography of the mid-dorsal surfaces of JFW and JBW colony eggs by AFM and SEM. AFM images from G70 JFW eggs in (**a**) 2D and (**c**) 3D; G70 JBW eggs in (**b**) 2D and (**d**) 3D. SEM images of (**e**) G73 JFW and (**f**) G73 JBW eggs. CT-central tubercle; EN-exochorionic network; OCC-outer chorionic cell; PT-peripheral tubercle. Ten µm scale bars are shown.

Representative 2D and 3D AFM images from G70 JFW and JBW colony eggs illustrate this topographical difference (Fig. 2a–d). SEM of G73 JBW and JFW colony eggs showed the presence of an extensive reticulated network termed the exochorionic network (EN) composed of hexagonal outer chorionic cells (OCCs)^30^. The OCC contains a central tubercle (CT) and several smaller peripheral tubercles (PTs) as previously observed in *Ae. aegypti* eggs^30^ (Fig. 2e&f with additional details in Supplementary Fig. 3). However, JBW colony eggs tended to have larger CTs and a more prominent EN than JFW eggs. The AFM and SEM findings therefore showed consistent differences between JBW and JFW colony egg surfaces, with the more prominent EN and CTs observed by SEM in JBW colony eggs likely contributing to their greater surface roughness and hardness compared to JFW colony eggs shown by AFM.

### Changes in larval cuticle surface topography also characterize salinity tolerance in *Aedes aegypti* laboratory colonies

**Figure 3:**
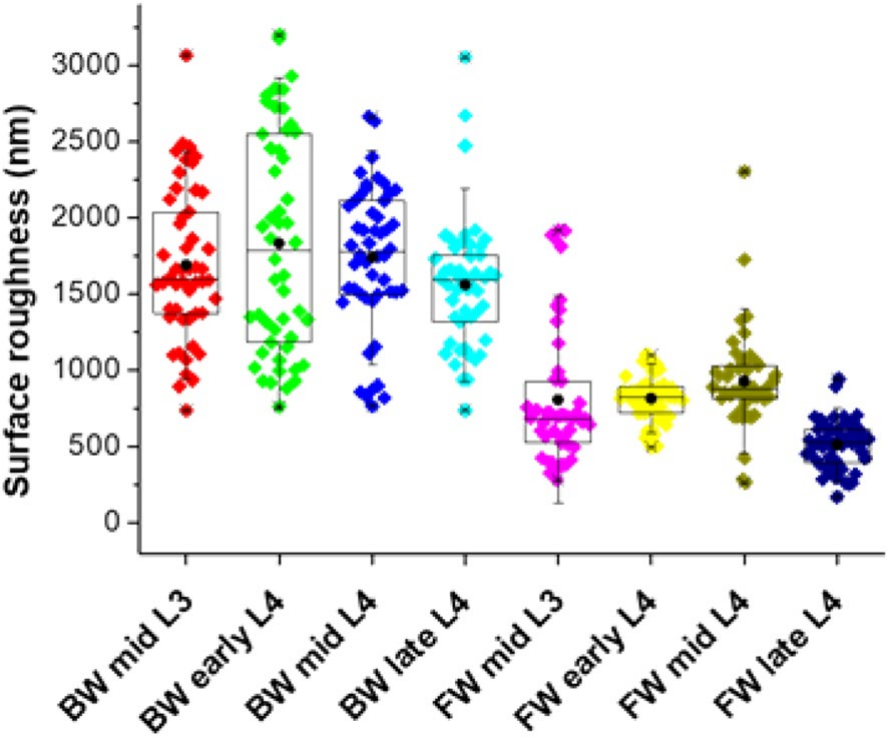
Surface topography of L3 and L4 cuticles from G79 JFW (labelled as FW in the figure) and JBW (labelled as BW in the figure) colonies by AFM. Surface roughness of the larval cuticles were determined as R_RMS_ in nm. Box-and-whiskers plots show the lower and upper quartiles and the median. Black solid circles show the mean.

Larvae that develop from *Ae. aegypti* eggs have four developmental stages (L1–L4) and transition between larval stages involves shedding of the old cuticle and synthesis of a new cuticle in the moulting process. Because larval cuticles are pliable structures, we were only able to determine their surface topography or roughness and not hardness or elasticity by AFM. This was done on mid-L3 through to late-L4 stage larvae from G79 JBW and JFW colonies. The cuticle surface roughness of JBW *Ae. aegypti* was greater than JFW *Ae. aegypti* in all corresponding larval stages from mid-L3 to late-L4 (Fig. 3).

Surface roughness was significantly higher in cuticles from the different stages of JBW larvae compared with cuticles from the corresponding stages of JFW larvae (Table 1). Surface roughness was lower in late-L4 than early or mid-L4 in both colonies which may be associated with preparation for pupation.

**Table 1:** Surface topography of L3 and L4 cuticles from JFW and JBW colonies by AFM.

Larval stage	Surface topography R_RMS_ in nm (mean ± SD)	
	JFW	JBW
Mid-L3	^B,^ ^C^ 809 ± 322	^A^1690 ± 309
Early-L4	^B,^ ^C^ 817 ± 96	^A^1835 ± 483
Mid-L4	^B^930 ± 266	^A^1739 ± 299
Late-L4	^C^514 ± 97	^A^1562 ± 288

Legend to Table 1: Surface topography of cuticle of larval stages that do not share a letter are significantly different (*p* < 0.05) from others in the same row and column. SD, standard deviation of the mean.

**Figure 4:**
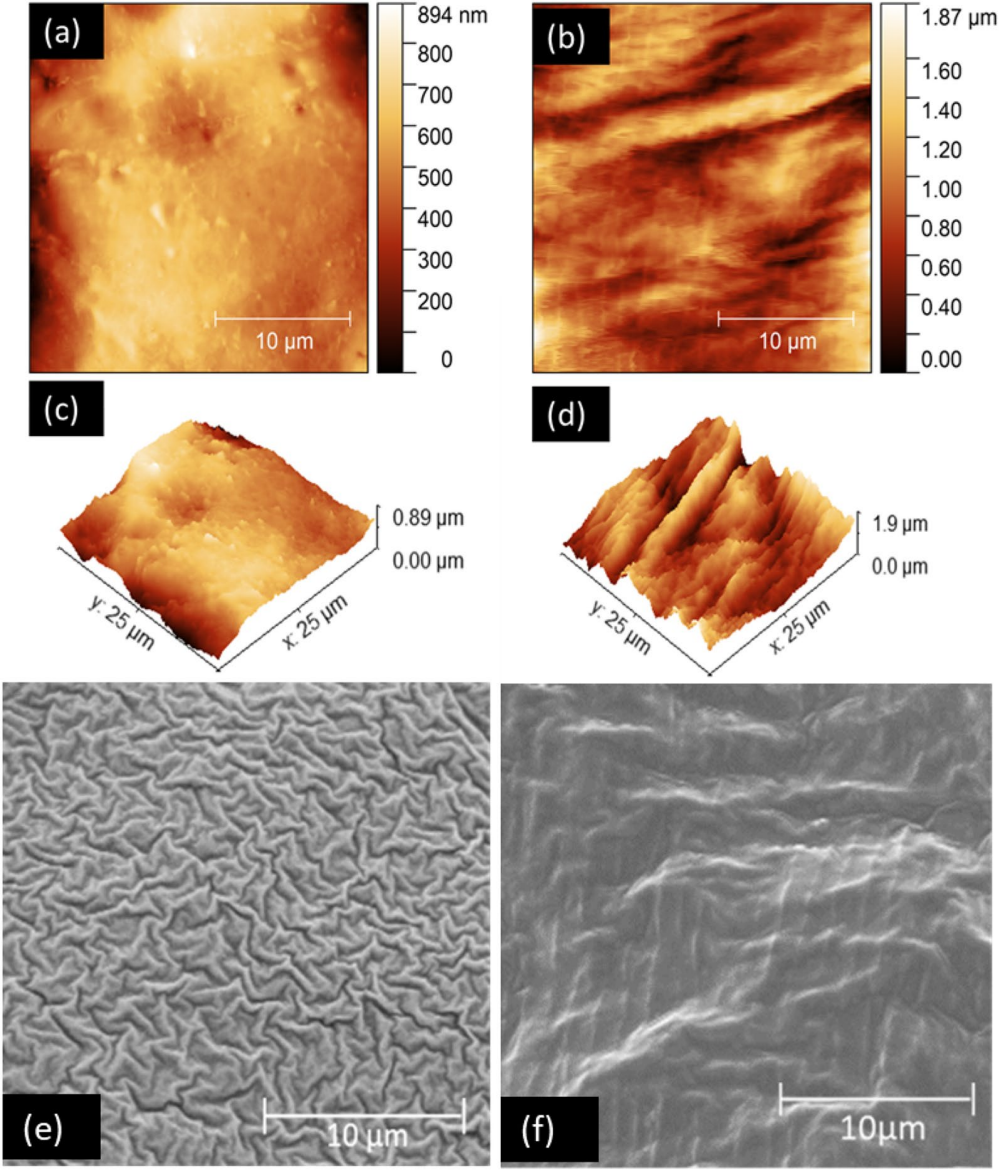
Surface topography of mid-L4 stage cuticles from JBW and JFW colonies by AFM and SEM. AFM images of cuticles from (**a**) G70 JFW and (**b**) G70 JBW in 2D, and (**c**) G70 JFW and (**d**) G70 JBW in 3D. Values shown are R_RMS_ in nm. SEM images of cuticles from mid-L4 of (**e**) G73 JFW and (**f**) G73 JBW. Ten µm scale bars are shown.

2D and 3D AFM images from mid-L4 cuticles from the JBW and JFW colonies shown in Fig. 4a–d illustrate the measured differences. Corresponding SEM images of mid-L4 cuticles of JBW and JFW colonies are shown in Fig. 4e–f. SEM of the mid-L4 JFW cuticle showed a uniform wrinkled appearance over the cuticle (Fig. 4e). In comparison, the mid-L4 cuticle from the JBW colony exhibited a more irregular surface with more prominent peaks and valleys (Fig. 4f). The findings with SEM are therefore consistent with AFM observations showing greater surface roughness in mid-L4 cuticles of the JBW colony compared to the JFW colony.

### Surface topography of *Aedes aegypti* L4 collected from BW and FW field habitats in the Jaffna peninsula

To determine whether field *Ae. aegypti* populations reflected the larval topography observed by AFM in the JBW and JFW laboratory colonies, we analyzed L4 collected from five inland FW containers and two coastal BW containers in the peninsula by AFM. The R_RMS_ values for surface cuticle topography of L4 were normally distributed within each container which was consistent with the L4 being derived from eggs laid by different single females in each container. The highest R_RMS_ was observed in L4 from a cement water tank in coastal Passaiyoor containing BW of 7 g/L salt (R_RMS_ 2025 ± 99 nm, mean ± standard deviation) and the lowest in a discarded plastic yoghurt pot in inland Thavady containing FW of 0 g/L salt (R_RMS_ 454 ± 84), with intermediate mean R_RMS_ values in L4 from the other five containers (Supplementary Table 3). The range of mean R_RMS_ values in field collections (Supplementary Table 3) was comparable to the range observed in different L4 stages from the JBW and JFW laboratory colonies (Table 1).

### Reduced susceptibility to temephos characterizes salinity tolerance in *Aedes aegypti* larvae

Because of the cuticle differences observed between JBW and JFW colony larvae in this study and previous proteomic and ultrastructural studies^21^, we investigated the susceptibility of JBW and JFW L3 and L4 to temephos, which is the most widely-used larvicide worldwide for *Ae. aegypti* source reduction. We determined the LC_50_ and LC_99_ of larvae over a period of 24 h to different dilutions of a stock temephos solution in the JBW and JFW (in G74–75) as well as the NFW (in G28–30) laboratory colonies. We additionally tested temephos susceptibility in (i) JBW *Ae. aegypti* that had been transferred to FW designated the brackish water reversal colony or BWR (in G11–13), and (ii) JFW laboratory *Ae. aegypti* that had been transferred to BW correspondingly designated FWR (in G11–13).

Results of the bioassays with L3 and L4 from the five *Ae. aegypti* laboratory colonies are shown in the Table 2. The LC_99_ was higher than the corresponding LC_50_ for L3 and L4 in all five colonies. L4 were less susceptible to temephos than the corresponding L3 in all five colonies, which is consistent with thicker cuticles being developed by L4 in preparation for pupation. L3 and L4 from the JFW and NFW colonies were significantly more susceptible to temephos than corresponding stages from the JBW colony. In L3 and L4 of the two colonies where salinity conditions had been reversed, viz*.,* BWR and FWR, the temephos susceptibility differed significantly from the parent JBW and JFW colonies, and approached the susceptibilities of larvae raised for a long time in the reversed salinity conditions, *i.e.,* JFW for the BWR larvae and JBW for the FWR larvae. However, significantly lower LC_50_ and LC_99_ persisted in FWR *Ae. aegypti* L4 compared with JBW *Ae. aegypti* L4 at 11–13 generations after the transfer from FW to BW (Table 2), showing that the greater susceptibility of JFW *Ae. aegypti* to salinity is an inheritable characteristic.

**Table 2:** Susceptibilities of L3 and L4 from the laboratory colonies to temephos over 24 h.

Colony	L3 (24 h)		L4 (24 h)	
	LC_50_ (95% CI) mg/L	LC_99_ (95% CI) mg/L	LC_50_ (95% CI) mg/L	LC_99_ (95% CI) mg/L
JBW	0.016^a^ (0.015- 0.017)	0.032 ^a^ (0.030–0.033)	0.038^a^ (0.036–0.039)	0.077^a^ (0.074–0.079)
JFW	0.007^b^ (0.005–0.008)	0.015 ^b^ (0.011–0.019)	0.012^c^ (0.008–0.016)	0.029^c^ (0.018–0.040)
NFW	0.006^b^ (0.006- 0.007)	0.016 ^b^ (0.014–0.017)	0.012^c^ (0.010–0.013)	0.028^c^ (0.028–0.029)
BWR	0.008^b^ (0.007–0.010)	0.019 ^b^ (0.016–0.021)	0.015^c^ (0.011–0.019)	0.032^b,^ ^c^ (0.019–0.045)
FWR	0.014^a^ (0.012–0.015)	0.031^a^ (0.029–0.034)	0.023^b^ (0.021–0.024)	0.049^b^ (0.046–0.053)

Legend to Table 2: LC_50_ – temephos concentration that kills 50% of larvae; LC_99_—temephos concentration that kills 99% of larvae. LC values between populations within each column were compared by Tukey’s method. Populations that do not share a letter are significantly different (*p* < 0.05). from others in the same column. CI, confidence interval; L3, third instar lava; L4, fourth instar larva. JBW, JFW, NFW, BWR, FWR—Jaffna brackish water, Jaffna fresh water, Nawalapitiya fresh water, brackish water reversal and fresh water reversal colonies respectively.

While temephos solution is sprayed on non-potable water habitats of *Ae. aegypti* near human dwellings*,* temephos 1% w/w absorbed on sand granules (temephos 1% SG) is commonly applied to potable FW in household containers^1–4^. Therefore, we also tested the susceptibility of *Ae. aegypti* L3 from the same five laboratory colonies to temephos 1% SG. Findings from L3 bioassays with the five *Ae. aegypti* colonies with temephos 1% SG are shown in Supplementary Table 4. The LC_50_ and LC_99_ for JBW *Ae. aegypti* L3 were significantly higher than the corresponding values for L3 from the JFW, NFW and BWR colonies. L3 from the JFW, NFW and JBW colonies had a similar pattern of relative susceptibilities to temephos 1% SG and temephos solution but with lower LC_50_ and LC_99_ for temephos 1% SG (Table 2 and Supplementary Table 4).

### Larval susceptibility to *Bacillus thuringiensis israelensis* (*Bti*) toxins

*Bacillus thuringiensis* var *israelensis* (*Bti*) produces several crystalline protoxins during sporulation which when ingested by mosquito larvae are activated into toxins that act through midgut receptors^37^. Because temephos acts in a different manner by inhibiting acetylcholinesterase in the neuromuscular system^38^, we also investigated the effects of *Bti* on JBW and JFW larvae. The average 24 h LC_50_ and LC_99_ of JBW L4 tended to be higher than JFW L4, but the differences were not statistically significant. The range of values for 24 h LC_99_ (0.014–0.022 mg/L) for L4 from G78–79 of JBW and JFW *Ae. aegypti* colonies (Supplementary Table 5) were an order of magnitude lower than the estimated recommended application rate for the *Bti* formulation of 0.3 to 0.7 mg/L, suggesting that *Bti* remains effective against BW and FW *Ae. aegypti*.

## Discussion

We utilized AFM for the first time to explore the topography and hardness (elasticity) in the outer surfaces of eggs as well as L3 and L4 larvae from two laboratory colonies of *Ae. aegypti* that had been adapted to 10 g/L salt (JBW colony) and 0 g/L salt (JFW colony). Our AFM findings showed that (i) JBW eggs possess greater surface hardness and roughness than JFW eggs, and (ii) JBW L3 and L4 cuticles exhibited more pronounced surface roughness than the corresponding JFW L3 and L4 cuticles.

SEM has previously been applied to study egg surfaces for a variety of purposes in different species of mosquitoes^30–35,39,40^, including *Ae. aegypti*^30,32,40^. We applied SEM for the first time to examine surface changes in mosquito eggs and larvae resulting from adaptation to salinity. Our SEM findings showed that the outer egg surface or chorion in both JFW and JBW *Ae. aegypti* contains an extensive reticulated network, termed the EN^30^. The EN is composed of hexagonal OCCs containing a large CT and several smaller PTs bordering the EN, as previously described in FW *Ae. aegypti*^30,32^. However, our results showed that JBW eggs possessed a more prominent EN and larger CTs than JFW eggs, which probably contribute to the greater surface roughness in JBW eggs detected by AFM. Furthermore, SEM of JBW L4 showed more raised cuticle surface corrugations than JFW L4, which is consistent with greater surface roughness of JBW L4 measured by AFM.

The EN has been reported to serve a protective function in the chorion and promote adherence to the egg laying surface^30,31^, and the CTs to facilitate gas exchange^33^. The chorion of mosquito eggs also protects them against desiccation^41^. The survival of *Ae. aegypti* eggs in BW requires adaptation to reduce water loss by osmotic efflux and the influx of Na^+^ and Cl^-^ ions. G2 and G5 eggs from a JBW *Ae. aegypti* laboratory colony hatched better in 10 g/L BW than JFW *Ae. aegypti*^19^. This was confirmed in the present study with G74 and G75 eggs and the greater hatchability in BW was further shown to be maternally inherited. The altered surfaces of JBW eggs, demonstrated by AFM and SEM are likely to be associated with their greater salinity tolerance and better hatchability in BW. The physiological and molecular mechanisms underlying the observed surface changes in JBW *Ae. aegypti* eggs, however, remain to be elucidated.

*Aedes aegypti* from JBW and JFW laboratory colonies differed in several biological properties within two and five generations after collection from the field, including salinity tolerance of preimaginal stages^9,19^, oviposition preference^19^, egg hatchability^19^, and preimaginal development periods^19^. RNAseq analysis of G28 JBW and G31 JFW generations of *Ae. aegypti* laboratory colonies showed that their L4 differed in the levels of mRNA transcripts for many cuticle proteins^21^, particularly members of the RR-2 family, consistent with changes in L4 cuticle protein composition observed by proteomic analysis^21^. Transcriptomic analysis of L4 further suggested more abundant synthesis of waxy epicuticle components in JBW *Ae.* *aegypti*^21^. The present finding of surface topographical changes in L3 and L4 abdominal cuticles of JBW *Ae. aegypti* by AFM and SEM are consistent with structural changes previously observed by transmission electron microscopy, viz*.*, JBW L4 abdominal cuticles were thicker and tended to contain more internal lamellae formed from chitin and chitin-binding proteins than JFW L4 abdominal cuticles^21^.

The abdomen has a large proportion of the total external surface of mosquito larva. The present findings are consistent with our previous proposal that cuticle changes in JBW *Ae. aegypti* larval abdomens contribute to their greater salinity tolerance by reducing permeability to water and ions^21^. This assertion is amenable to further experimental investigation with appropriate techniques. Different physiological and structural adaptations to maintain larval haemolymph osmolarity have been described in mosquito species that naturally develop in BW^42–45^, but their larval cuticle structure has not been studied. The pupae of such naturally salinity-tolerant mosquito species, however, possess more thickened and sclerotized cuticles that are less permeable to water and ions than fresh water species^42^.

No reproductive barrier was observed between JBW and JFW *Ae. aegypti* in the G2 and G5 generations of laboratory colonies established with mosquitoes collected from BW and FW field habitats that were approximately 5 km apart^19^. We confirmed the absence of a reproductive barrier between the G74 and G75 of the JBW and JFW colonies. However, adaptation to BW in *Ae. aegypti* were shown to entail changes in egg hatching properties and adaptive genetic changes in larvae in G2 and G5^19^. Our present findings with JBW and JFW *Ae. aegypti,* including observations on the two colonies subject to a reversal of rearing salinity for 11–13 generations, also demonstrated salinity-adaptive genetic changes that manifest in egg and larval phenotypes. Our present and previous^9,19^ findings suggest that selection at the level of oviposition, egg hatching and larval development leads to genetic changes in *Ae. aegypti* adapted to BW.

No location in the 1100 km^2^ Jaffna peninsula is > 10 km from the sea or lagoon^24^ (Supplementary Fig. 1). The inland locations of Nallur, Thirunelvely and Thavady where L4 were collected from FW containers are respectively on average 2.5, 4.2 and 6.8 km away from coastal Passaiyoor where larvae were collected from BW containers (Supplementary Fig. 1). Several constraints apply to comparing cuticle topography in field L4 collected within the Jaffna peninsula with L4 from the JBW and JFW laboratory colonies. They include (i) the difficulty in classifying field-collected larvae as early, mid or late-L4 because they develop from eggs laid at different times in different containers; (ii) the absence of barriers to genetic exchange within wild *Ae. aegypti* populations in the relatively small Jaffna peninsula; (iii) dispersion of female *Ae. aegypti* seeking sites for oviposition variously estimated to be 0.5 to 3.6 km^46,47^ but probably further in the strong winds that prevail in the peninsula. Hence correlating surface egg and larval changes with salinity in field habitats is better investigated in *Ae. aegypti* populations separated by greater distances than is possible in the Jaffna peninsula.

Larval source reduction by eliminating or reducing preimaginal habitats and applying larvicides is critical for controlling the transmission of dengue by its primary vector *Ae. aegypti* and the secondary vector *Ae. albopictus*^1–4^. *Aedes aegypti* is anthropophagic and *Ae. albopictus* is partly anthropophagic^1^ and both can feed on humans during the day and outdoors^1–3,48^^.^ Hence indoor residual spraying of insecticides and insecticide impregnated bed nets, used for controlling indoor feeding and resting *Anopheles* malaria vectors, are less effective against the two *Aedes* vectors^4^. Dengue control efforts by the Ministry of Health in the Jaffna peninsula include applying temephos 1% w/w SG to potable water in domestic wells and water storage tanks as well as other FW collections within domestic premises. Epidemics of dengue are controlled by applying an emulsifiable temephos concentrate using compression sprayers to non-potable FW preimaginal habitats near human dwellings, and thermal fogging with pyrethroid insecticides to target adult vectors.

*Bacillus thuringiensis* var *israelensis* and *Lysinibacillus sphaericus* containing crystalline inclusions of protoxins in spores are also used as environmentally safe and effective larvicides against mosquito vectors^37^. The protoxins are activated on ingestion to form toxins that bind to midgut receptors to disrupt midgut function and cause larval death^37^. Temephos kills larvae in a different manner by inhibiting acetylcholinesterase and disrupting neuromuscular function^38^. Temephos is likely to enter the larval body by ingestion and, because of its lipophilic nature, through the cuticle as well. A cuticle covers the external surface of the larva and is also present in the tracheal lumen and the gut with the exception of the midgut. Thicker cuticles leading to reduced penetration of pyrethroid insecticides in adult anopheline mosquito vectors are associated with pyrethroid resistance^49–51^. Changes in the external cuticles of JBW *Ae. aegypti* larvae reported here may therefore contribute to their greater resistance to temephos than JFW larvae. Alterations observed in the midgut transcriptome of BW *Ae. aegypti* larvae^21^ may also contribute to their greater resistance to temephos through other mechanisms. Changes in the midgut or external cuticle of JBW *Ae. aegypti* larvae, however, do not confer significantly greater resistance to the midgut-acting biological larvicide *Bti*.

Artificial selection with temephos that increases the levels of different enzymes that catabolise temephos can produce temephos resistance in *Ae. aegypti* larvae^52^ but there is no evidence that such mechanisms or acetylcholinesterase mutations^38^ cause the greater resistance seen in JBW *Ae. aegypti*. The WHO guidelines regard values for LC_99_ > 0.012 mg/L in larval bioassays to reflect temephos resistance. The LC_99_ of L3 and L4 therefore classifies the JBW *Ae. aegypti* laboratory colony as resistant to temephos. This is also the case for JFW and NFW colonies when tested with temephos solution but not for the JFW colony with temephos 1%SG. However, the JBW and JFW colony larvae and wild *Ae. aegypti* populations in the Jaffna peninsula are expected to be susceptible to higher concentrations of temephos (up to 1 mg/L) permitted for use in potable water. Our findings also show that the recommended application rates for *Bti* will be effective against BW and FW *Ae. aegypti* larvae in the peninsula.

*Aedes albopictus* also exhibits greater tolerance to salinity for preimaginal development to adults in the Jaffna peninsula than mainland Sri Lanka^9^. The responsible genetic and physiological mechanisms have not been investigated in *Ae. albopictus*. However, the salinity-adaptive changes we observe in *Ae. aegypti* are also likely to occur in *Ae. albopictus*. Our findings suggest that in coastal zones it is (i) essential to extend larval source reduction efforts to BW habitats, and (ii) important to continue monitoring the efficacy of widely-used larvicides.

## Methods

### JBW and JFW laboratory colonies of *Aedes aegypti*

Self-mating JBW and JFW laboratory colonies of *Ae. aegypti* initiated with larvae collected from BW and FW habitats in the Jaffna peninsula (Supplementary Fig. 1), as previously described^9,19^ were used for experiments. JFW and JBW *Ae. aegypti* larvae were respectively maintained in potable tap water obtained from an artesian well in Thirunelvely (Supplementary Fig. 1), and sea water diluted with the tap water to yield 10 g/L salt. Because FW *Ae. aegypti* in the Jaffna district were more tolerant of salinity than FW mosquitoes from the mainland of Sri Lanka^9^, we also initiated a laboratory colony termed NFW with *Ae. aegypti* larvae collected from Nawalapitiya in the central hills of mainland Sri Lanka (Supplementary Fig. 1), where mosquitoes are not exposed to BW habitats. The NFW colony was maintained in tap water purified by reverse osmosis in the insectary of the Department of Zoology, University of Jaffna. Larvae in colonies were reared in 30 × 25 cm plastic trays containing 1.5 L water with up to 150 larvae per tray and fed with fish meal powder twice a day. Adults and larvae were maintained at 28–30 °C at a relative humidity ~ 75% and 12 h dark and light periods. Adult mosquitoes were allowed to feed every 3 days on Balb/c mice and on 10% glucose pledgets provided at other times. Appropriate 10 g/L salt BW or 0 g/L salt FW surfaces for oviposition were provided in the cages after blood feeding, and eggs harvested 2 days later as previously described^19^.

### Establishment of reversal colonies to test larval susceptibility to temephos

To determine the effects of reversing the larval rearing salinity on temephos susceptibility, reversal colonies were independently initiated 63 generations (G63) after the initial establishment of JBW and JFW *Ae. aegypti* colonies. From the 10 g/L salinity JBW colony, a reversal colony (BWR) was established by collecting eggs on 0 g/L FW egg-laying surfaces and then hatching and rearing larvae in FW. Similarly, a FW reversal colony (FWR) was established from the 0 g/L JFW colony by collecting eggs on 10 g/L BW egg-laying surfaces and subsequently hatching and rearing larvae in 10 g/L BW.

### Determination of salinity tolerance for the L1 to adult transition

L1 from the G66 JFW, G66 JBW and G17 NFW colonies were exposed to salinities of 0, 2, 4, 6, 8, 10, 12, 15, 18 and 20 g/L as previously described^19^. The required salinities were obtained by diluting seawater with tap water and measuring salinity with a refractor salinometer (Atago, Japan). Forty larvae introduced in 300 ml of water of specific salinity in 500 ml plastic containers, were maintained at 28–30 °C, relative humidity ~ 75% and 12 h dark and light periods until adults emerged. Larvae were fed twice daily with powered fish meal. Test media were changed on alternate days. Three replicate tests were performed for each salinity level. Mortality of larvae, the numbers of emerging adults and LC_50_/LC_90_ for salinity were determined as previously described^19^.

### Determination of reproductive compatibility between *Aedes aegypti* laboratory colonies and egg hatchabilities

Adults from both G74 and G75 of the JFW and JBW laboratory colonies were separately tested for reproductive compatibility. Male and female pupae were differentiated morphologically as described^53^ and separated. Experiments were done with 1-day-old virgin male and female adults emerging from pupae. Fifteen JFW males were allowed to mate with 15 JBW females and similarly 15 JFW females were crossed with 15 JBW males. Control crosses were performed with 15 JFW males and 15 JFW females as well as 15 JBW males and 15 JBW females. After 3–4 days of mating, the females were fed on Balb/c mouse blood with 10% glucose pledgets provided at other times. Two days later, each cage was provided with an egg-laying surface moistened with water to reflect the rearing salinity of the original colony of crossed female mosquito, *i.e.,* cages with JFW females and JBW females were respectively provided with 0 g/L and 10 g/L salt egg laying surfaces. After 2 days, the egg cups were removed from the cages and the number of laid eggs in each cup determined. From each egg cup, 100 eggs were collected and allowed to hatch in water with either 0 g/L or 10 g/L salt. The number of the 100 eggs hatching into larvae (hatchability) and then transforming in adults were determined from each set of 100 eggs. Larvae were fed twice daily with powdered fish meal.

### *Aedes aegypti* eggs and larvae from JBW and JFW laboratory colonies for AFM

Single female derived eggs (24–30 h after oviposition) and randomly selected mid-L4 (36–40 h post-ecdysis) progenies from the G70 of JFW and JBW colonies and, randomly selected mid-L3 (36–40 h post-ecdysis), early-L4 (18–24 h post-ecdysis), mid-L4 (36–40 h post-ecdysis) and late-L4 (65–70 h post-ecdysis) larvae from the G79 JBW and JFW colonies were used for AFM.

### *Aedes aegypti* L4 collected from BW and FW containers in the field for AFM

*Aedes* larvae were collected during April and May 2022 from four sites in the Jaffna peninsula (Supplementary Fig. 1): Passaiyoor—a coastal fishing area along the Jaffna lagoon with FW and BW sources (9° 38′ 49.4″N, 80° 1′ 58.8″E), Thirunelvely – an inland location with FW sources (9° 41′ 1.9″N, 80° 1′ 18.7″), Thavady—an inland location with FW sources (9° 42′ 32.42″N, 80° 0′ 50.9″E) and Nallur—an inland location with FW sources (9° 40′ 21.16″N, 80° 1′ 50.1″E). Preimaginal stages were collected from small containers with 5 ml plastic pipettes^6^. Ten dips with string-connected conical drop nets were used for cement water storage tanks^6^. Salinity of larval habitats was measured with a hand-held refracto-salinometer (Atago Co. Ltd., Tokyo, Japan). Collected larvae were brought to the insectary of the Department of Zoology, University of Jaffna, and morphologically identified as *Ae. aegypti* using published keys^54^, with L4 stages being further characterized as described^55^.

*Aedes aegypti* L4 were collected from five discarded FW containers outside houses in inland areas: plastic flower pot (Nallur), two plastic yoghurt pots (Thirunelvely and Thavady), a plastic bowl (Nallur) and a coconut shell (Nallur). All contained rain water. *Aedes aegypti* L4 were also collected from two cement water storage tanks with 6 and 7 g/L salt BW in coastal Passaiyoor. The tanks stored water pumped from nearby BW wells, and the stored water then used for washing fishing nets. Field-collected *Ae. aegypti* L4 were directly processed for AFM analysis as described below.

### Atomic force microscopy

A Park XE7 AFM machine (Park Systems, Suwon, South Korea) was used to record images with Park SmartScan (1.0RTM12b.B20) software in windows OS. Specimens were washed in distilled water, air dried, firmly fixed to the metallic sample holder with double-sided adhesive tape and placed on the sample stage. Images were captured by non-contact mode scanning with PPP-NCHR cantilevers (Park systems, Suwon, South Korea) at 0.1 Hz with 256-pixel lines, and processed with Park Systems XEI (4.3.4 build 22) software and Gwyddion v2.59^56^.

Surface topography variation was estimated by root mean square averages for roughness (R_RMS_) which measures the average height deviations^57^. R_RMS_ in nm was computed by squaring the measurements of the heights of protuberances and depressions, averaging the squares, and calculating the square root of that number using Park Systems XEI (4.3.4 build 22) software (Park Systems, Suwon, Korea). AFM images were captured by non-contact mode scanning a 25 µm × 25 µm area in the center of intact eggs. All FW eggs were first analyzed by AFM followed by all BW eggs. Measurements for R_RMS_ were made on two randomly selected 5 µm × 5 µm areas using Gwyddion v2.59. A total of thirty eggs from six *Ae. aegypti* females (comprising five eggs per female) were analyzed resulting in 60 readings each for the G70 JFW and G70 JBW colonies.

For R_RMS_ measurements on larvae, they were washed three times in distilled water and placed on filter paper to absorb excess water and left to dry for 2–3 min. Each larva was then firmly fixed to the metal AFM sample holder with double-sided adhesive tape and placed on the sample stage. AFM images were captured by non-contact mode scanning a 25 µm × 25 µm area of the dorsal region of the mid-6th abdominal segment of randomly selected intact mid-L4 from five larval trays (two larvae per tray) of the G70 JFW and G70 JBW colonies. AFM was performed sequentially first on all FW samples and subsequently all BW samples. The same procedure for AFM was then applied to randomly selected mid-L3, early-L4, mid-L4 and late-L4 from the G79 JBW and JFW colonies. From each 25 µm × 25 µm area AFM image, R_RMS_ measurements were taken from five randomly selected 5 µm × 5 µm areas using Gwyddion v2.59, resulting in 50 readings for each colony. The R_RMS_ of all 20 L4 collected from FW and all 10 L4 from BW field habitats were measured by the same procedure.

The modulus of elasticity (Young’s modulus) of egg surfaces was measured in the cantilever bending mode by AFM, where the AFM probe is used to apply a vertical force^58^. The same Park XE7 AFM machine (Park Systems, Suwon, South Korea) was used to record force-distance (FD) curves with Park SmartScan (1.0RTM12b.B20) software in windows OS. PPP-CONSTSCR tips with a tip radius of curvature of less than 10 nm and a force constant of 0.2 Nm^-1^ were used. A tip diameter of 10 nm was assumed. The cantilever was moved towards the sample surface for a certain distance, while the cantilever vertical deflection was recorded^58^. The modulus of elasticity was calculated as the ratio of the loading force to the distance travelled by the cantilever tip. The force-distance curves from a FW and BW sample are shown in Supplementary Fig. 4. The modulus of elasticity values in mega Pascals (MPa), which is equivalent to 10^6^ Nm^-2^, were obtained with Park Systems XEI (4.3.4 build 22) software and using a Hertzian model for four-sided pyramid tips. Two measurements were done on randomly selected 5 µm × 5 µm areas on five eggs from six females from each of JFW and JBW colonies.

### Scanning electron microscopy

Eggs (24–30 h after oviposition) and mid-L4 (36–40 h post-ecdysis) from G73 of JFW and JBW colonies were used for scanning electron microscopy (SEM). Specimens were immersed in 2.5% glutaraldehyde (Sigma-Aldrich, MO, USA) in 0.1 M sodium cacodylate buffer pH 7.3 (Sigma-Aldrich, MO, USA) and kept for 24 h at 4 °C. After three washes in sodium cacodylate buffer (pH 7.3), specimens were post-fixed overnight at 4 °C in 1% osmium tetroxide in sodium cacodylate buffer (pH 7.3). This was followed by dehydration sequentially through ethanol at 30%, 50%, 70%, 90% concentration (5 min each) and then three times for 10 min in 100% ethanol. The specimens were washed twice with 1 ml Hexamethyldisilazan (HMDS) and mounted on double-sided carbon tabs on aluminum stubs. Specimens were then air-dried and sputter-coated with gold for 25 s to create a homogenous surface over the samples and to increase secondary electron signals. Finally, specimens were viewed in a Zeiss Gemini 450SEM (Jena, Germany) and images captured at 5.00 kV. Five mid-L4 from JBW and JFW colonies were examined, and at least two images obtained for each sample in the middle region of the sixth abdominal segment. Similarly, a minimum two different regions of the middle of dorsal surface of five eggs from the JFW and JBW colonies were photographed.

### Light microscopy of eggs and mid-L4 from JBW and JFW colonies

Eggs and mid-L4 larvae from JFW and JBW *Ae. aegypti* laboratory colonies were examined by light microscopy. The maximum length and width of single eggs were measured in an Olympus CX21 light microscope equipped with an ocular micrometer with 0.01 mm least count under 100 × magnification. One hundred eggs from ten iso-females (comprising ten eggs per iso-female) were analyzed per colony with measurements replicated for three different generations (G62, G66, G70). Measurements from the three generations per colony were pooled (total 300 readings) and Tukey’s method for multiple comparisons in the analysis of variance was used to compare the egg length and width of the populations at 95% CI.

### Larval bioassay to determine susceptibility to temephos solution

The susceptibility of larvae from the five laboratory colonies at different generation: JFW (G74–75), JBW (G74–75), NFW (G28–G30), FWR and BWR (G11–13) to temephos dissolved in absolute ethanol containing 2% butanone (Vector Control Research Unit, School of Biological Sciences, Universiti Sains Malaysia, Gelugor, Malaysia) was determined using standard WHO bioassay conditions^59^. Eight different concentrations of temephos test solutions (0.001–0.4 mg/L) were prepared by diluting the 156.25 mg/L temephos stock solution with tap water (0 g/L salt). A group of 20 early-L3 larvae (18–24 h post-ecdysis) were placed into the 500 ml plastic cups filled with 125 ml test solution. Each concentration of temephos was tested in four replicate larval bioassays. Control larvae were treated with 0.5 mg/L butanone present at the highest concentration of temephos used in the assay. The bioassays were similarly performed under the same conditions with early-L4 (18–24 h post-ecdysis) from the five colonies. Each bioassay was repeated three times. All the bioassays were performed under standard laboratory conditions (temperature 28–30 °C, relative humidity ~ 75%). Larval mortality after 24 and 48 h of exposure were recorded.

Because temephos is commonly applied absorbed on sand granules (SG) to potable FW in containers, we also determined the susceptibility of L3 to temephos SG formulation with 1% w/w temephos as the active ingredient (Anti-Malaria Campaign, Health Ministry, Sri Lanka) in JFW (G66-69), JBW (G66-69), NFW (G20-23), FWR (G5-8) and BWR (G5-8) laboratory colonies. Fifty early-L3 (18–24 h post-ecdysis) were treated with a series of temephos SG at levels giving 2–100% mortality at 24 h. Varying amounts of finely-powdered temephos SG were evenly applied to 20 × 30 cm plastic trays containing of 2 L of FW and larvae introduced into each tray 15 min later. Three replicates were performed for each of eight temephos1% SG levels tested per bioassay. A control group was tested with FW alone. Larval mortality after 24 and 48 h of exposure was recorded. Each bioassay was repeated three times with larvae from three different colony generations. Fewer than 10% of larvae pupated in controls and therefore all bioassays were considered valid according to the WHO guidelines^60^. Larval assay results were analysed using the log-probit method^61^ as described below in the section on statistical analyses.

### Larval bioassay to determine susceptibility to *Bacillus thuringiensis israelensis* (*Bti)* toxin

Mosquito Bits® (Summit® Responsible Solutions, Baltimore, USA), a *Bti* granular formulation containing spores and insecticidal toxins, was used to test the susceptibility of L4 from the JFW (G78–79) and JBW (G78–79) laboratory colonies. The manufacturer recommends its application at 113–227 g per 93 m^2^ of water surface to eliminate mosquito larvae from field habitats. The fine powder was dispersed in tap water (0 g/L salt) at 0.1 mg/L. This was held for 30 min at ambient temperature while stirring with a glass rod and then filtered through 12 µm Whatman filter paper. The stock was diluted with tap water seven different concentrations (0.001–0.016 mg/L) that gave 0–100% mortality. A batch of 25 early L4 larvae (18-24 h post-ecdysis) was introduced in 2 L plastic trays containing 1 L test solution. Four replicates were tested at each concentration together with a tap water control. The numbers of dead larvae were determined 24 h and 48 h after exposure to *Bti* and each bioassay was repeated twice.

### Statistical analyses

The Shapiro–Wilk normality test was performed in order to test for normal distribution of values in all data sets.

Tukey’s method for multiple comparisons in the analysis of variance (ANOVA) was performed to compare group means of percent hatchability and percent survival to adulthood in experiments to determine reproductive compatibility. Tukey’s method for multiple comparisons in the analysis of variance was used to compare the egg length and width of the colonies at 95% CI.

Two-tailed Student’s t-tests were performed to determine the significant differences in modulus of elasticity (measured in MPa) and surface roughness (measured as nm R_RMS_) of eggs from the JFW and JBW colonies. Means were calculated for every individual larva of the laboratory and field samples and the significance of differences in means among life stages, different field habitats and laboratory-reared single female progenies were compared by Tukey’s method for multiple comparisons in the analysis of variance (ANOVA) at 95% CI.

Larval assay results were analysed by the log-probit method^61^ using Minitab 17 software (Minitab LLC, State College, PA, USA) to estimate the slope of regression lines and determine the 50% lethal concentration (LC_50_) and 99% lethal concentration (LC_99_) with 95% confidence intervals (CIs). The LCs determined for temephos larval bioassay for each colony was the average of the three bioassays. Tukey’s method for multiple comparisons in the analysis of variance (ANOVA) was used to compare the LCs of the test colonies at 95% CI. LC values for JBW and JFW colonies from the two separate *Bti* bioassays were pooled for comparison by the two-tailed Student’s t-test.

### Ethics approval

Mosquitoes were collected and reared in the insectary as per the approved (AERC/2014/02) protocol of the Institutional Animal Ethics Committee of the University of Jaffna.

## Supplementary Information

Supplementary Information.

## Data Availability

All data supporting the conclusions of this article are included within the article and its supplementary files.
