# Supplementary material for: Resistance to the larvicide temephos and altered egg and larval surfaces characterize salinity-tolerant Aedes aegypti
Source: Sci Rep. 2023 May 19;13:8160. doi: 10.1038/s41598-023-35128-1 (PMC10198600; doi:10.1038/s41598-023-35128-1)
Supplement: Supplementary file 1 — Supplementary Information. [file 41598_2023_35128_MOESM1_ESM.docx]

**Supplementary information**

**Resistance to the larvicide temephos and altered egg and larval surfaces characterize salinity-tolerant *Aedes aegypti***

**Supplementary Figure 1.** **Map of Sri Lanka with relevant locations**

**
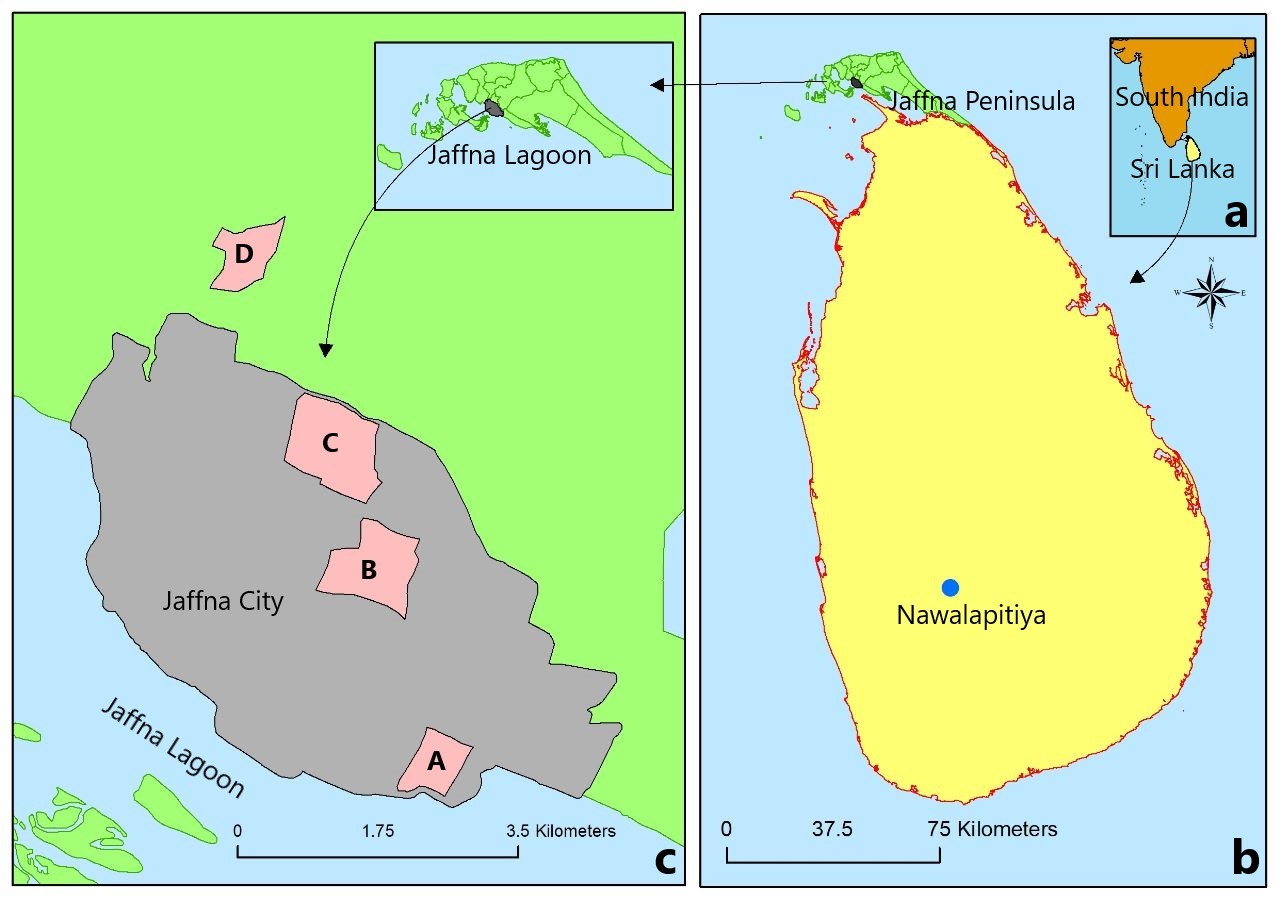
**

**a)** The geographical location of Sri Lanka in close proximity to South India in the Indian ocean; **b)** map of Sri Lanka showing the location of Nawalapitiya in the central highlands with exclusively freshwater habitats and the Jaffna peninsula; **c**) Jaffna city and locations where L4 were collected in the field. A – Passaiyoor, B – Nallur, C – Thirunelvely and D – Thavady. Scale bars show distances.

**Supplementary Figure 2: Light microscope images of JFW and JBW *Aedes aegypti***

**
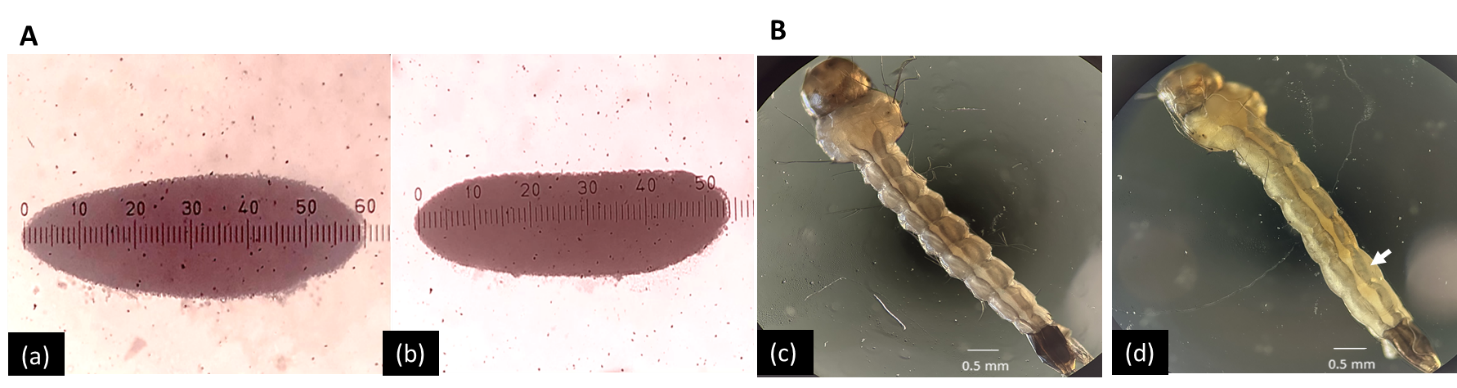
**

**C**

| **Colony** | **Maximum length (mm)** | **Maximum width (mm)** |
| --- | --- | --- |
| JFW | 0.61±0.03 | 0.17±0.02 |
| JBW | 0.57±0.02 | 0.16±0.01 |

**A.** **Light microscope images of G73 JFW and JBW *Ae. aegypti* eggs**: (a) JFW and (b) JBW eggs. Samples were photographed in an Olympus CX21 microscope incorporating an ocular micrometer at 100X magnification. The central surface region of intact eggs was used for AFM and SEM. **B**. **Light microscope images of G73 JFW and G73 JBW *Ae. aegypti* mid-L4 stages**: (c) JFW and (d) JBW mid-L4 showing the dorsal view of head, thorax and abdominal segments. L4 were viewed in an Olympus CX21 microscope and photographed at 10X magnification. The dorsal region of the mid-6th abdominal segment (arrowed in d) were used for AFM and SEM. Scale bars are shown. **C.** **Dimensions of JFW and JBW colony eggs**: Measurements from three generations (G62, G66, G70) per colony were pooled (total 300 readings) and Tukey’s method for multiple comparisons in the analysis of variance was used to compare maximal egg length and width of JFW and JBW eggs at 95% CI. The results are shown as mean ± standard deviation. The maximum lengths and widths of JFW and JBW eggs were significantly different at p < 0.05.

**Supplementary Figure 3: Additional SEM images of eggs from G73 JFW and JBW *Aedes aegypti* laboratory colonies**

**
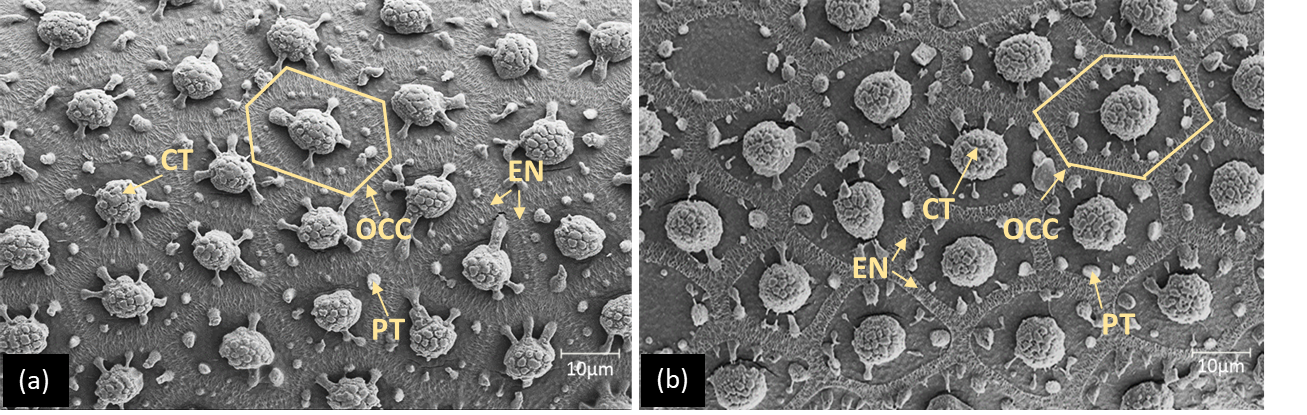
**

(**a**) JFW (G73) and (**b**) JBW (G73) eggs (110 x 75 µm surface area). Mid-dorsal surfaces of eggs were observed in a Gemini 450SEM, ZEISS microscope at 5 kV. CT- central tubercle, EN- exochorionic network, OCC- hexagonal outer chorionic cell, PT- peripheral tubercle. Scale bars are shown.

**Supplementary Figure 4.** **Force-distance curves for determining Young’s modulus (YM) of egg surfaces**

**
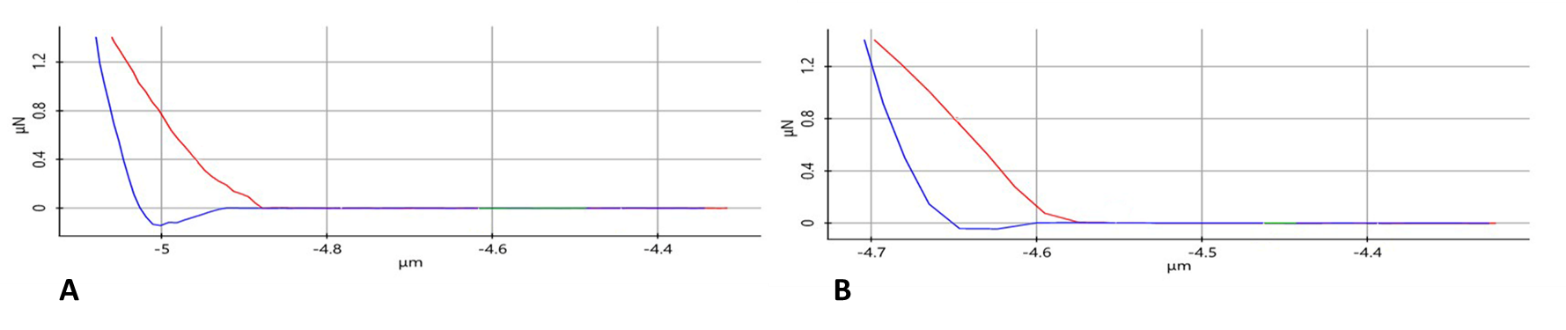
**

**A**. Results from a G70 JFW egg (YM: 29 MPa) and **B**. G70 JBW egg (144 MPa) are shown. The Hertzian model for the four-sided pyramid tips was used to calculate hardness from the force-distance curves. The red and blue lines respectively show the approaching and retracting curves.

**Supplementary Table 1. Salinity tolerance of the JFW, JBW and NFW *Aedes aegypti* laboratory colonies**

| Colony (Generation) | LC_50_ in g/L salt | 95% CI of LC_50_ |
| --- | --- | --- |
| NFW (G17) | 11.2 | 10.3-12.2 |
| JFW (G66) | 11.6 | 10.7-12.4 |
| JBW (G66) | 16.0 | 14.9-17.1 |

LC_50_ for salt in the transition from L1 to adults in the G17 NFW colony, and G66 JFW and JBW colonies are shown. Generations shown are since colony establishment from field collected larvae^19^. CI – confidence interval. LC_50_ was calculated with 95% confidence intervals using the Minitab 17 statistical software. Confidence intervals that do not overlap demonstrate significant differences at *p*<0.05 between the two FW colonies and the JBW colony.

**Supplementary Table 2. Reproductive compatibility of JBW and JFW laboratory colonies and preimaginal development of resulting eggs**

| Experiment | JFW **♂** X JBW **♀** | JFW **♀** X JBW **♂** | JFW **♀** X JFW **♂** | JBW **♀** X JBW **♂** |
| --- | --- | --- | --- | --- |
| Percent eggs hatching in 0 g/L salt | ^a^91± 0.7 | ^a^86 ±5.6 | ^a^91 ±2.1 | ^a^84 ±8.4 |
| Percent eggs hatching in 10 g/L salt | ^a^88 ±2.1 | ^b^62 ±5.6 | ^a,b^ 74±6.3 | ^a^88±6.3 |
| Percent survival to adulthood in 0 g/L salt | ^a^89 ±1.4 | ^a^85 ±4.9 | ^a^90 ±0.7 | ^a^82±8.4 |
| Percent survival to adulthood in 10 g/L salt | ^a,b^85 ± 0 | ^c^59 ± 5.6 | ^b,c^65 ±6.3 | ^a^86 ±5.6 |

The mean percent hatchability and mean percent survival to adulthood between crosses were compared by Tukey’s method. Results are means ± standard deviations of the mean. Values that do not share a letter are significantly different (*p* < 0.05) from others in the same row.

**Supplementary Table 3. Surface topography of cuticles from *Aedes aegypti* L4 collected in FW and BW field habitats**

| **FW collections** | | | **BW collections** | | |
| --- | --- | --- | --- | --- | --- |
| **Habitat type and location (salinity)** | **Number of larvae measured by AFM** | **Surface roughness (R_RMS_ mean ± SD in nm)** | **Habitat type and location (salinity)** | **Number of larvae measured by AFM** | **Surface roughness (R_RMS_ mean ± SD in nm)** |
| Plastic flower pot, inland Nallur (0 g/L) | 3 | ^c^482 ± 104 | Cement water tank-1, coastal Passaiyoor (6 g/L) | 5 | ^c^633 ± 57 |
| Plastic yoghurt pot, inland Nallur (0 g/L) | 4 | ^c^661 ± 224 | Cement water tank-2, coastal Passaiyoor (7 g/L) | 5 | ^a^2025 ± 99 |
| Plastic yoghurt pot, inland Thavady (0 g/L) | 5 | ^c^454 ± 84 |  |  |  |
| Plastic bowl, inland Thirunelvely (0 g/L) | 5 | ^b^1293 ±124 |  |  |  |
| Coconut shell, inland Nallur (0 g/L) | 3 | ^b^1595 ± 390 |  |  |  |

R_RMS_ values were normally distributed within individual containers due probably to the larvae therein being derived from eggs laid by single females. Mean values that do not share a letter are significantly different (*p* < 0.05) from others in the same row and column. SD – standard deviation of the mean. The locations of collection sites in the peninsula are shown in Supplementary Figure 1.

**Supplementary Table 4. Susceptibilities of *Aedes aegypti* L3 from different colonies to temephos 1% SG after 24 hours of exposure.**

| Population | LC_50_ mg/L temephos (95% CI) | LC_99_ mg/L temephos (95% CI) |
| --- | --- | --- |
| JBW | ^a^0.0043 (0.0040 - 0.0045) | ^a^0.0147 (0.0138-0.0156) |
| JFW | ^b^0.0011 (0.0008 - 0.0013) | ^b^0.0081 (0.0051- 0.0110) |
| NFW | ^b^0.0016 (0.0010 - 0.0021) | ^b^0.0084 (0.0047- 0.0121) |
| BWR | ^b^0.0017 (0.0015 - 0.0018) | ^b^0.0078 (0.0069- 0.0086) |
| FWR | ^a^0.0036 (0.0035 - 0.0036) | ^a^0.0122 (0.0116- 0.0128) |

CI – 95% confidence interval of the mean; LC values between populations within each column were compared by Tukey’s method. Results that do not share a letter are significantly different (*p* < 0.05) from others in the same column.

**Supplementary Table 5. Susceptibilities of L4 from laboratory colonies of JBW and JFW *Aedes aegypti* to *Bacillus thuringiensis* var *israelensis* (*Bti*) toxin**

| Colony | LC_50_ mg/L *Bti* at 24 h  (95% CI) | LC_99_ mg/L *Bti* at 24 h  (95% CI) |
| --- | --- | --- |
| JBW (G78) | 0.008 (0.007 - 0.009) | 0.022 (0.019- 0.028) |
| JBW (G79) | 0.007 (0.006 - 0.008) | 0.017 (0.015- 0.021) |
| JFW (G78) | 0.007 (0.006 - 0.008) | 0.019 (0.017 -0.024) |
| JFW (G79) | 0.005 (0.004- 0.006) | 0.014 (0.012 -0.017) |
|  | t=1.341  *p*=0.311 | t=0.848  *p*= 0.485 |

CI – confidence interval**.** Results of two tailed Student’s t-tests do not show a significant difference in sensitivity to *Bti* between the Jaffna FW and BW *Ae. aegypti* colonies.
